# Autoimmune neutropenia associated with influenza virus infection in childhood: a case report

**DOI:** 10.1186/s12879-021-06506-9

**Published:** 2021-08-18

**Authors:** Ignacio Callejas Caballero, Marta Illán Ramos, Arantxa Berzosa Sánchez, Eduardo Anguita, José Tomás Ramos Amador

**Affiliations:** 1 Department of Paediatrics, Hospital Universitario Clínico San Carlos, Madrid, Spain; 2 Department of Hematology, Hospital Universitario Clínico San Carlos, IML, IdISSC, Madrid, Spain; 3 Department of Medicine, UCM, Madrid, Spain

**Keywords:** Autoimmune neutropenia, Childhood, Influenza infection, Case report

## Abstract

**Background:**

Although neutropenia is relatively frequent in infants and children and is mostly a benign condition with a self-limited course, it can lead to life-threatening severe infections.

Autoimmune neutropenia is a relatively uncommon hematological disorder characterized by the autoantibody-induced destruction of neutrophils. It is usually triggered by viral infections with very few documented cases after influenza virus.

**Case presentation:**

An 8-month-old male infant presented at the emergency room with a 5-days history of fever up to 39.7 °C, cough and runny nose.

In the blood test performed, severe neutropenia was diagnosed (neutrophils 109/μL). A nasopharyngeal aspirate revealed a positive rapid test for Influenza A.

Serum antineutrophil antibodies were determined with positive results. Neutropenia targeted panel showed no mutations. Despite maintenance of severe neutropenia for 9 months the course was uneventful without treatment.

**Conclusions:**

When severe neutropenia is diagnosed and confirmed, it is essential to rule out some potential etiologies and underlying conditions, since the appropriate subsequent management will depend on it.

Although autoimmune neutropenia triggered by viral infections has been widely reported, it has seldom been reported after influenza infection.

The benign course of the disease allows a conservative management in most cases.

## Background

The diagnosis and management of neutropenia in children is a real challenge for pediatricians. The differential diagnosis is complex and there is a potential risk of serious complications, including mortality in some cases.

The cause of neutropenia can be primary or secondary to a broad spectrum of underlying diseases, including immunodeficiency, rheumathological disease, lymphoproliferative disorders, solid tumors or drug-induced.

In the pediatric age, primary autoimmune cytopenia with a single line cell involvement are much more frequent than those with a secondary origin. Autoimmune thrombocytopenia and hemolytic anemia are much more common than autoimmune neutropenia. The involvement of more than one cell line is usually due to a predisposing secondary cause warranting a more extensive diagnosis work-up.

Autoimmune neutropenia is usually a benign process starting more frequently in toddlers, in which the autoimmune nature is infrequently determined. Furthermore, the triggering etiology is seldom recognized. There is little information regarding the autoimmune cause due to the difficulty in diagnosis and very scarce data on the triggering etiology.

It is usually triggered by viral infections [1–4] with very few documented cases after influenza virus [5]. We present here the case of an infant diagnosed of autoimmune neutropenia after infection by the Influenza A virus.

## Case presentation

An 8-month-old male infant presented at the emergency room in March 2020 with a 5-days history of fever up to 39.7 °C, cough, runny nose and occasional vomiting.

The mother reported mild asthenia and partial decrease in oral intake.

He did not attend daycare. He did not receive any drug but paracetamol in the current episode to control the fever. The patient was still breast-fed and the mother reported mild cough and runny nose with no fever (or flu-like symptoms).

Personal and family history was unremarkable. There was a controlled pregnancy, delivering by caesarian section at 39 weeks of gestational age. No resuscitation was required and no infectious risk factors were identified.

The neonatal period was uneventful with normal weight gain and development. Vaccinations were up to date according to the Community of Madrid calendar, including also rotavirus and 4CMenB without remarkable side-effects. The patient was exclusively breast-fed during the first 6 months of life, with adequate weight gain, and normal stool consistency. There was no history of mouth ulcers or relevant infectious episodes.

No previous blood tests were available, since the patient had never before undergone blood drawn.

On physical exam at the emergency room, the temperature was 36.9 °C, oxygen saturation 98% with room air and heart rate 155 bpm. The child was in good general condition, well hydrated and perfused with no signs of respiratory distress. The mouth was normal without sores, ulcers or exudates. The lung auscultation revealed mild hypoventilation in the left base.

The rest of the physical exam was considered normal.

In the emergency department, a chest radiograph was performed, showing diffuse retrocardiac consolidation. Blood tests were requested with the following parameters:

Leukocytes 7800/μL (neutrophils 109/μL, 1.4%; lymphocytes 6630/μL, 85.2%; monocytes 1030/μL, 13.2%; eosinophils 7.8/μL, 0.1%; basophils 7.8/μL, 0.1%). The biochemical tests revealed normal glucose, creatinine and aminotranferases. Both C-reactive protein (CRP) and procalcitonin (PCT) were within normal limits (CRP < 0.29 mg/dL, PCT 0.06 ng/mL).

A nasopharyngeal aspirate obtained at the emergency room revealed a positive rapid test for Influenza A and negative for RSV. The blood culture was drawn and turned out to be negative.

After diagnosing the severe neutropenia, the mother told us the infant had adequate height and weight gain, without relevant infections or alterations in stools. No drugs in the last days-weeks were given, but paracetamol in the current episode. He did not present dysmorphic features. No family history of autoimmunity was reported. The patient was discharged from the emergency room with a diagnosis of neutropenia and influenza A infection and referred to the outpatient clinic for further evaluation.

Once neutropenia was confirmed 1 month after diagnosis, hemograms were performed every 7–10 days in the following month, in which the number of neutrophils was always between 100 and 400/μL. There was no anemia or thrombocytopenia. Immunoglobulins levels, vitamin B12, and folic acid were within the normal range for age.

SARS-CoV2 serology 1 month after going to the emergency room resulted negative. In addition, serologies to CMV, EBV, parvovirus B19, HIV, *Mycoplasma pneumoniae* and *Chlamydia pneumoniae* were also negative.

Granulocyte-specific antibodies were detected by flow cytometric assay and Luminex-based multiplex fluorescent bead platform (LABScreen™ Multi, One Lambda Thermo Fisher Scientific) at 10 and 16 months of age for detection against HLA-Class I, Class II and HNA Antigens.

The child remained asymptomatic at all hospital visits. During the 14 months of follow up since diagnosis the patient has had an uneventful course with normal growth and development. He has completed vaccinations including MMR, chickenpox and influenzae without complications. At the age of 14 months he started daycare having two mild upper respiratory episodes, one of them associated with COVID-19 confirmed by nasopharyngeal aspirate PCR and positive serology.

A thorough genetic NGS panel for congenital neutropenia, including the following genes was performed by DNA target sequence enrichment capture with Miseq illumine analysis, being all unmutated: *AK2*, *AP3B1*, *BLOC1S3*, *CDAN1*, *CLPB*, *COH1/VPS13B*, *CSF3R*, *CXCR4*, *DTNBP1/BLOC1S8*, *DYN2/DNM2*, *EIF2AK3/PERK*, *ELANE/ELA2*, *G6PT1/SLC37A4*, *G6PC3*, *GATA-1*, *GFI1*, *HAX1*, *HPS1*, *HPS3*, *HPS4*, *HPS5*, *HPS6*, *JAGN1*, *LYST*, *MAPBPIP/LAMTOR*2, *MYO5A*, *PLDN/BLOC1S6*, *RAB27A*, *RMRP/CHH/NME1*, *SEC23B*, *SLC19A2*, *SLC25A38*, *SLC37A4*, *SMARCAL1*, *TAZ*, *TCIRG*1, *USB1*, *VPS13B*, *VPS45*, *WAS/SCNX*.

At the age of 21 months, a blood count showed that the patient had recovered the neutrophil count (total ANC 1100/μL).

## Discussion and conclusions

Influenza A as a trigger for neutropenia has been reported so far in just a few cases [5].

Our case report has shown the association of an influenza A infection in a healthy infant with autoimmune neutropenia. Although a causal relationship cannot be established, it is postulated that influenza A infection might have triggered the development of severe neutropenia, subsequently found to be autoimmune.

Neutropenia is relatively common in childhood and poses a diagnostic challenge. In general terms, benign chronic neutropenia presents with > 500 neutrophils/μL, whereas autoimmune neutropenia appears with < 500 neutrophils/μL. In cases of severe neutropenia, an extensive work up is frequently started resulting in burdening investigations and unnecessary treatment. It is therefore important to attempt to reach the etiological diagnosis in cases with < 500 neutrophils/μL.

As we have seen, it may be necessary to study possible triggering infections in the context of severe neutropenia, usually of viral origin, that might help in sparing unnecessary medical evaluation or even invasive procedures like bone marrow biopsy.

The microbiologic work up to be done might be of major importance in order to perform the appropriate microbiological tests (rapid tests, multiple PCRs, serologies, etc.) that may be associated with the severe neutropenia.

The association of a possible viral trigger does not preclude from excluding other causes of severe neutropenia. In these cases, a close clinical and analytical follow-up with serial extractions is usually warranted, to rule out entities such as cyclic neutropenia or the involvement of other cell lines (red and platelet series).

Autoimmune neutropenia is a benign disease typically presenting in infants or toddlers. In a large series of 240 children with autoimmune neutropenia the median age of onset was 8 months, all of them presenting before 3 years of age [6], although the presentation may be beyond that age in early childhood [7]. The course is variable, with spontaneous resolution in most cases that may last several months or years. In two large series the median times of resolution were 17 months and 3 years, respectively [6, 7]. Although sometimes a genetic panel to detect the mutation of common genes associated with congenital neutropenia is performed [8], it is not usually necessary when there is a high suspicion of autoimmune neutropenia [7]

Children with autoimmune neutropenia rarely suffer from serious infections, since circulating neutrophils, although low in number, are normally functional, and therefore capable of defending the host against such infections. Furthermore, the severity and type of infections are poorly related with the neutrophil count. It is important to investigate other findings that may rise the suspicion of an underlying diagnosis, including dysmorphic features, hepatosplenomegaly, bone pain or chronic diarrhea. They tend to develop only minor infections of the upper respiratory tract, skin and soft tissues. By contrast, children who develop serious bacterial or fungal infections should be studied in greater depth to rule out immunodeficiencies and other causes of neutropenia. Although exceptional, severe infections have been reported in children with autoimmune neutropenia. Pyogenic liver abscesses in an 8-month-old boy and a 9-month-old girl diagnosed of autoimmune neutropenia have recently been reported [9]. There have been other isolated cases of neck abscesses [10] and persistent perianal abscesses [11] related to autoimmune neutropenia. In these severe cases a complete work-up to rule out immunodeficiencies should always be considered [9].Therefore, we should bear in mind that autoimmune neutropenia, although rare, can be associated with severe infections, including abscesses.

The diagnosis of autoimmune neutropenia is often difficult, due in part to the poor sensitivity and specificity of antineutrophil antibody tests [12]. If only a single type of testing method is used, the positive rate is lower. Instead, if several testing methods are used, the diagnosis will more likely be correct. It is therefore advisable to perform the autoantibody study by two different methods to establish the diagnosis. Indirect neutrophil immunofluorescence tests and agranulocyte agglutination tests are the most frequently used methods.

Direct granulocyte test finds out antibodies embedded on the patient’s neutrophils and may give false positives results. This could be produced by a low number of isolated granulocytes or unspecific attachment of immunoglobulin G (IgG) immune complexes to the Fcγ receptors.

On the other hand, false negative results are less common so we could reasonably rule out the disease with these tests.

Indirect test detects a reaction between donor neutrophils and free granulocyte-specific antibodies in the patient’s serum. On the contrary to direct test, it shows a minor false positive rate and a high proportion of false negatives. Its sensitivity is limited by the possible absence of the full human neutrophil antigens pattern in the neutrophil suspension and low levels of autoantibodies.

The limitations of direct and indirect tests make it difficult to reach a steady diagnosis of autoimmune neutropenia and often the condition suffered by these children is classified as ‘chronic/idiopathic benign neutropenia of infancy’, although many of these infants are likely to have autoimmune neutropenia.

An important diagnostic clue is monocytosis, as it is usually present in autoimmune neutropenia. However, in most cases bone marrow morphology is not informative; even though normal/hyper cellular marrow, generally with a late maturational arrest, may support the diagnosis.

Unlike other disorders with severe congenital neutropenia or entities associated with secondary neutropenia (HIV, some primary immunodeficiencies, anatomic or functional asplenia, chemotherapy or post-transplantation), most patients do not require preventive treatment, beyond adequate oral hygiene and mouthwashes. Children who have recurrent infections or a previous surgical procedure may benefit from granulocyte colony stimulating factor (G-CSF) [8]. Some clinicians have suggested the potential benefit of prophylaxis with broad spectrum antibiotics. Nevertheless, there is no evidence of the benefit of this approach and side effects and resistance issues should be balanced in autoimmune neutropenia, even when the absolute neutrophil count is very low. Although it was common practice in the past [6], routine antibiotic prophylaxis is not usually recommended nowadays [13, 14].

In our case we did not give any prophylaxis, nor antibiotic treatment when the patient presented with fever, but we closely observed the patient at the emergency room through baseline blood tests.

One limitation of this report is the fact that our patient had no previous blood counts, since a previous normal blood count would rule out congenital neutropenia. For this reason, a thorough genetic panel to study congenital neutropenia was performed with negative results, supporting our diagnosis of autoimmune neutropenia.

We describe an influenza-related autoimmune neutropenia in an infant. Other causes of neutropenia have been ruled out and the likely triggering etiology has also been identified.

Until now, there are very few reported cases of autoimmune neutropenia triggered by influenza virus infection.

Our case highlights the importance of adequately targeting neutropenia since proper subsequent management will depend on it. Even when severe neutropenia is diagnosed, the documentation of a viral infection as a possible triggering etiology and the demonstration of the autoimmune nature in an infant allow a conservative management, and reassuring the family about the benign course of the disease and its spontaneous resolution over time.

## Data Availability

All relevant data generated or analysed during this study are included in this published article. The complete data is registered in the electronic hospital´s medical record. If you needed more data of the patient we would provide it from the electronic hospital´s medical record.

## Abbreviations

CRP: C-reactive protein
COVID-19: Coronavirus disease 2019
EVB: Epstein-Barr virus
G-CSF: Granulocyte colony stimulating factor
HLA: Human leukocyte antigen
HNA: Human neutrophil antigens
HIV: Human immunodeficiency virus
MMR: Measles, mumps, rubella
NGS: Next generation sequencing
PCR: Polymerase chain reaction
PCT: Procalcitonine
RSV: Respiratory syncytial virus
SARS-CoV-2: Severe acute respiratory syndrome coronavirus 2
